# DNA Fingerprinting and Species Identification Uncovers the Genetic Diversity of Katsouni Pea in the Greek Islands Amorgos and Schinoussa

**DOI:** 10.3390/plants9040479

**Published:** 2020-04-09

**Authors:** Evangelia Stavridou, Georgios Lagiotis, Lefkothea Karapetsi, Maslin Osathanunkul, Panagiotis Madesis

**Affiliations:** 1 Institute of Applied Biosciences, Centre for Research and Technology, Thermi, GR-570 01 Thessaloniki, Greece; estavrid@certh.gr (E.S.); glagiotis@certh.gr (G.L.); lefki8@certh.gr (L.K.); 2 Department of Biology, Faculty of Science, Chiang Mai University, Chiang Mai 50200, Thailand; maslin.cmu@gmail.com; 3 Research Center in Bioresources for Agriculture, Industry and Medicine, Chiang Mai University, Chiang Mai 50200, Thailand

**Keywords:** pea landraces, Amorgos, Schinoussa, DNA Barcoding, ISSR genotyping, HRM analysis, powdery mildew

## Abstract

Pea (*P. sativum* L.), one of the most important legume crops worldwide, has been traditionally cultivated in Lesser Cyclades since ancient times. The commonly known traditional pea cultivar, ‘Katsouni’, is endemic to the islands of Amorgos and Schinoussa and is of great local economic importance. Despite the widespread cultivation of ‘Katsouni’ in both islands, it is still unknown whether the current Schinoussa and Amorgos pea populations are distinct landraces, and if they have common evolutionary origin. To assist conservation and breeding of the pea crop, the genetic diversity and phylogenetic relationships of 39 pea samples from Amorgos and 86 from Schinoussa were studied using DNA barcoding and ISSR marker analyses. The results indicate that both populations are different landraces with distinct geographical distribution and are more closely related to *P. sativum* subsp. *elatius* than the *P. abyssinicum* and *P. fulvum* species. Further characterization of the ‘Katsouni’ landraces for functional polymorphisms regarding pathogen resistance, revealed susceptibility to the powdery mildew (*Erysiphe pisi* DC.). This work represents the first investigation on the genetic diversity and population structure of the ‘Katsouni’ cultivar. Exploiting the local genetic diversity of traditional landraces is fundamental for conservation practices and crop improvement through breeding strategies.

## 1. Introduction

Species in the economically important *Fabaceae* family have been a staple human food for millennia and their use is closely related to human evolution [1]. Legumes, such as Spanish vetchling (*Lathyrus clymenum* L.), lentils (*Lens culinaris* M.) and beans (*Phaseolus vulgaris* L.) are an important plant-based protein source, rich in mineral nutrients, complex starch and fibers, and contain health-promoting antioxidants, such as carotenoids and phenolic compounds [2,3,4]. The usage of leguminous crops in traditional crop rotation systems, reduces the need for synthetic nitrogen-based fertilizers by forming symbiotic relationships with nitrogen (N)-fixing soil bacteria [5]. Such management practices are of great ecological importance and have high potential for conservation agriculture, considering legumes are functional either as growing crop or as crop residue [6]. 

Pea (*P. sativum* L.) is among the most important legume crops, such as chickpea (*Cicer arietinum* L.), lentil and faba bean (*Vicia faba* L.), in temperate climates and has a wide geographical distribution, with field pea being specifically adapted to a wide range of climates and altitudes. The *Pisum* species are of high commercial importance and are cultivated worldwide for dry and fresh consumption. According to the International Legume Database (ILDIS) and to the classification of Maxted and Ambrose (2001) [7], the *Pisum* genus includes three species: i) *Pisum abyssinicum*, ii) *Pisum fulvum* and iii) *Pisum sativum* L., which further includes the wild pea, *Pisum sativum* subsp. *elatius* (M. Bieb. Asch. & Graebn) and the domesticated pea, *Pisum sativum* subsp. *sativum*. 

Phylogenetic analyses of various pea taxa with molecular markers indicate that hybridization between wild peas is not an extensive phenomenon [8]. The recently annotated pea genome sequence and the resequencing of data from 42 wild, landrace and cultivar *Pisum* genotypes, provided further insights into legume genome evolution [9]. It has been suggested that the common ancestor of the *Pisum* species was probably cytogenetically similar to *P. sativum* subsp. *elatius*, which evolved across the Mediterranean and Middle East [8] and gave rise to *P. sativum* subsp. *sativum* and *P. fulvum* in the northern Middle East. Regarding *P. abyssinicum*, two main hypotheses exist; it is considered the result of a domestication event from a southern *P. sativum* subsp. *elatius* ancestor [10] followed by a migration to Abyssinia, possibly through ancient human trading routes [11], indicating at least two domestication events independent of *P. sativum* subsp. *sativum* [9,12]. The alternative hypothesis about the origins of *P. abyssinicum* suggests that it derived from a hybridization event between *P. fulvum* and *P. sativum* subsp. *elatius*, which occurred in the western half region of the Fertile Crescent [13] and then a small sample was introduced to north-eastern Africa, where it evolved into the modern *P. abyssinicum* [14]. The very low genetic diversity in the Abyssinian pea suggests that the taxon has recently experienced a severe bottleneck or is a relatively young taxon [10] and the hybridization event has most likely occurred about 4000 years bp [15].

The *Pisum* genus is very diverse, showing the gamut of relatedness that reflect taxonomic identifiers, eco-geography and breeding gene pools [8,12,16,17]. Several phenotypic classification studies on pea germplasm are based on agronomical characteristics and morphological descriptors [18,19,20,21], which are unreliable for the evaluation of pea genetic resources and the identification of different cultivars in the Fabaceae family [22], especially considering the environmental effects on the expression of the genotype. Several different molecular methods have been previously employed to assess the genetic diversity in the *Pisum* genus, such as Random Amplification of Polymorphic DNA (RAPD) [23,24,25], Inter-Single Sequence Repeats (ISSRs) [23,24,26], Single Sequence Repeats (SSR) [27,28], Retrotransposon-Based Insertion Polymorphism (RBIP) markers [13,17,29,30,31] and Expressed Sequence Tags (EST)-derived genomic markers [32]. Additionally, high-throughput parallel genotyping via genome-wide next generation sequencing techniques have also been used to study the diversity of wild pea [8,33,34].

An alternative method for the simple and accurate authentication of plant species is DNA Barcoding. The *rbcL* and *matK* regions have been recommended as core DNA barcodes for plant identification [35]. In *Fabaceae* species, four coding chloroplast regions (*rpoB*, *rpoC1*, *rbcL*, and *matK*) and two non-coding nuclear regions (*ITS1* and *ITS2*) have been used as barcodes [22,36,37,38]. DNA barcoding has also been used to reconstruct the phylogenetic relationship of the main Mediterranean leguminous crops [39]. Furthermore, the combination of DNA barcoding with high resolution melting analysis (Bar-HRM) has, thus far, been proved an effective approach for the identification of diverse plant species, their Protected Designation of Origin (PDO) products and quantification of adulterants [40,41,42,43].

In Greece, pulses have been traditionally cultivated since the ancient times and is a staple food in the local culinary culture. A popular Greek dish (namely ‘Fava’) is typically prepared using different legume species, such as yellow- split peas (*P. sativum* L.) or faba beans (*Vicia faba*). However, in the island of Santorini, the authentic PDO ‘Fava Santorinis’, is exclusively prepared from a local grass pea variety of *L. clymenum*. In other Cycladic islands, especially in Amorgos and Schinoussa, ‘Fava’ is prepared from the dried peeled and split seeds of an endemic *Pisum* cultivar also known as ‘Katsouni’, named after the convex shape of a small sickle’s lobe used for mowing the crop. ‘Katsouni’ is a traditional product and a crop of great economic importance for the Lesser Cyclades. Currently, is in the process to be appointed as a PDO EU mark, offering a significant income to the local farmers. ‘Katsouni’ is fully adapted to the local climatic conditions of the Cyclades, with dry and hot summers and mild winters. It is rich in proteins (over 22%) and can be stored after drying the seeds throughout the year.

Historic records indicate that the ‘Katsouni’ landrace is the result of long-term selection and evolution from prehistoric times that occurred in Amorgos. Since the mid-19th century ‘Katsouni’ was transferred to the deserted Schinoussa island by residents of neighbouring Amorgos, who moved to settle there, and has since been cultivated uninterruptedly. However, Schinoussa was not always an abandoned island. Archaeological excavations have revealed findings indicating great activity from the prehistoric times to the Classical and Hellenistic period [44]. In the Byzantine times, trade and commerce were essential components of the island’s prosperity [45], which heralded an age of advancement, especially during the Venetian rule (13th–16th century). However, during the Ottoman rule (16th–19th century) the island was deserted as indicated by the famous botanist and traveler de Tournefort (2003) [46]. In the late Middle Ages, in 1537, Cyclades along with Schinoussa were plundered by the Ottoman pirate Haiderin Barbarossa [47] and piracy continued up to the 19th century [48]. With Schinoussa becoming a pirates’ den, we hypothesized that either: (i) the current Schinoussa pea population is an independent landrace, which is possibly the result of introgression of *P*. *abyssinicum* (transferred by pirates) into the *P. sativum* subsp. *elatius* germplasm; (ii) the Amorgos and Schinoussa populations belong to the same widely distributed landrace, or (iii) the two landraces emerged from the split of an ancestral population.

The knowledge of genetic relationships and diversity among individual landraces is fundamental for conservation practices and the selection of appropriate parents in breeding programs. Hence, in the present study, we evaluated the application of ISSR marker analysis and DNA barcoding for the molecular characterization of local Amorgos and Schinousssa pea populations. Furthermore, considering that powdery mildew (*E. pisi* DC.) severely affects pea crops worldwide [49], and in the frame of targeting functional polymorphisms, we aimed at characterizing the two landraces for the presence of the powdery mildew resistance gene (*er1-7)* with HRM analysis. The diversity assessment of local landraces may not only provide insights in understanding pea phylogenetics and population genetics, but also broaden pea breeding strategies.

## 2. Results

### 2.1. DNA Barcoding, Sequencing and Tree Analysis

To identify potential inter- and intra-specific variation between the two pea populations of Amorgos and Schinoussa, 24 pea samples were analyzed in the present study using the *ITS2*, *trnL* and *rpoC*, and 21 for the *psbA*-*trnH* and *matK* barcoding regions. The selection of *matK* and *psbA*-*trnH* was based on the unique SNPs observed in the aligned sequences among the three species and especially between *P. abyssinicum* and *P. sativum* (Figure S1). The amplification of *trnL* and *rpoC* regions was 100% successful, whilst for the *psbA-trnH*, *ITS2* and *matK* sequences the rate was 95.24%, 95.8% and 90.47%, respectively.

The positive amplicons were sequenced, and based on the BLAST results, most of the markers were able to identify the samples at the genus level, but not at the species level, when blasted against the NCBI database. The BLAST entries matched with all the three *Pisum* species (*P. sativum* subsp. *sativum*, *P. sativum* subsp. *elatius*, *P. fulvum* and *P. abyssinicum*), with over 96% similarity in identity and coverage. However, the *ITS2* marker identified all target sequences as *P. sativum* subsp. *elatius* with 100% similarity to the available sequences.

The sequences of each barcode gene were aligned and compared against indicative BLAST entries. In *psbA-trnH* we observed an intel polymorphism among our samples and the NCBI database entries. Most of our samples showed a gap in the barcoding region similar to that observed in the *elatius* subspecies sequences, but not in the *P. abyssinicum* and *P. fulvum* sequences (Figure S1). Other observed variations include the *P. abyssinicum*-specific polymorphism of a thymine (T) in place of a cytocine (C), and an unspecific SNP (G/T) present exclusively in the samples of both islands and the *P. sativum* subsp. *elatius* NCBI entries (Figure S1). Regarding the *ITS2* barcode, only the brown colored peas from both islands shared a common nucleotide variation of a guanine (G) in place of an adenine (A) (Figure S2). The *matK* barcoding showed a *P. fulvum*-specific polymorphism (C/A) and other unspecific SNPs (Figure S2). However, the observed variations in *trnL* and *rpoC* regions were not consistent among either seed coat color and/or landrace (Figure S2). Furthermore, both *matK* and *psbA-trnH* regions showed higher diversity and pairwise distance values compared to the other markers (Table S1); yet, the observed low genetic variation values across all barcoding regions (Table S1) indicate that the populations are closely related with a recent common ancestor.

**Figure 1:**
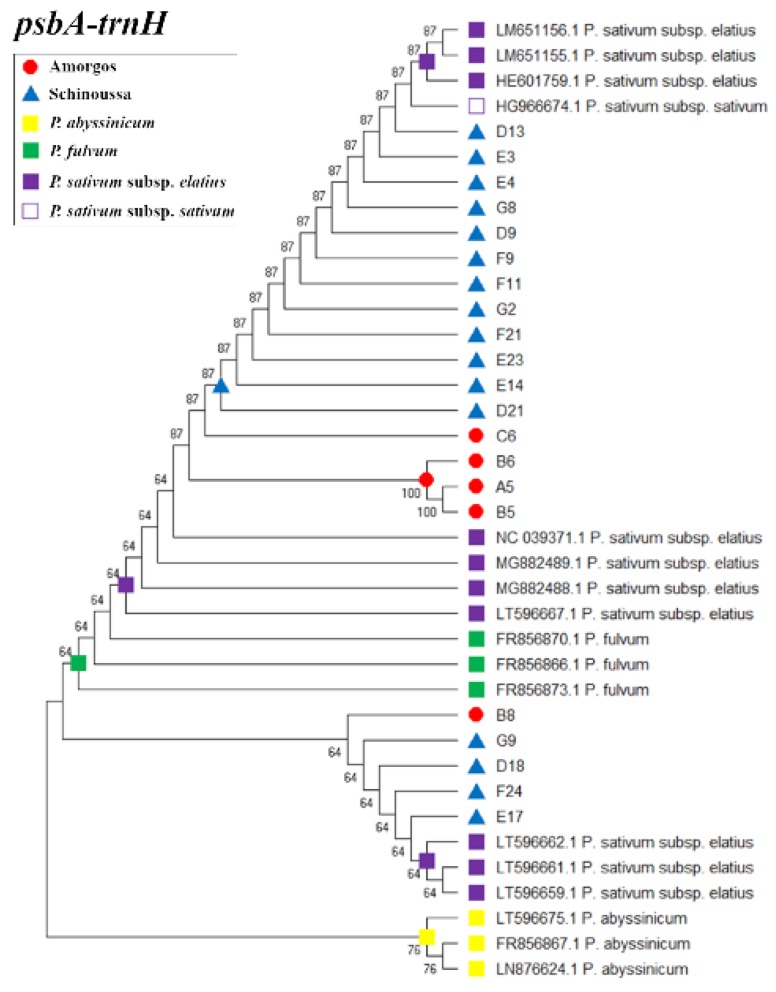
The *psbA-trnH* dendrogram illustrating the phylogenetic relationships between Amorgos (red circle) and Schinoussa (blue triangle) pea populations. The corresponding NCBI sequences of the *P. sativum* subsp. *elatius* (purple square), *P. sativum* subsp. *sativum* (white square), *P. abyssinicum* (yellow square) and *P. fulvum* (green square) were used as reference taxa.

To provide a basic illustration of the phylogenetic associations between the landraces, the DNA barcoding data was used to calculate the genetic distances and generate Neighbour-joining dendrograms (Figure 1 and Figure S3). The *psbA-trnH* dendrogram illustrates the clustering of the *P. abyssinicum* sequences supported by 76% bootstrap value, which are distinct from the rest of the samples at 64%, emphasizing that our samples probably do not belong to *P. abyssinicum* (Figure 1). In contrast, both the main structures of the *trnL* and *ITS2* dendrograms (Figure S3) separated the two landraces in two distinct clusters, which also corresponded to the represented geographical regions, although supported by a low bootstrap value (30–60%). The main structure of the *rpoC* dendrogram (Figure S3) presented also two clusters separating the pea taxa from Schinoussa to those of Amorgos island, supported by a higher bootstrap value (59–66%). However, the *matK* dendrogram (Figure S3) did not show any significant patterns. Taking into consideration the results from the barcoding analysis, the two populations probably belong to the *P. sativum* subsp. *elatius*.

### 2.2. ISSR Genotyping

To further investigate the genetic differences between the two populations, we used fifteen ISSR markers, out of which six were found to be polymorphic. ISSR analysis of the pea populations using the six polymorphic markers yielded 66 bands in total; five unique bands were identified for the Schinousasa population, whereas the remaining 61 bands were shared between the two populations (Table 1). The Schinoussa population presented a significantly greater polymorphism compared to Amorgos by displaying a greater number of different alleles (Na), whilst the number of effective alleles (Ne) did not show significant differences (Table 2).

The genetic differentiation between landraces (PhiPT distances) was significantly different (PhiPT = 0.188; P ≤ 0.001), indicating that the two landraces are geographically distinct. The Analysis of Molecular Variance (AMOVA) based on the PhiPT values indicated that most of the genetic diversity occurred within landraces (80%), while the variability among landraces contributed to the 20% of the observed genetic diversity (Table 3).

**Figure 2:**
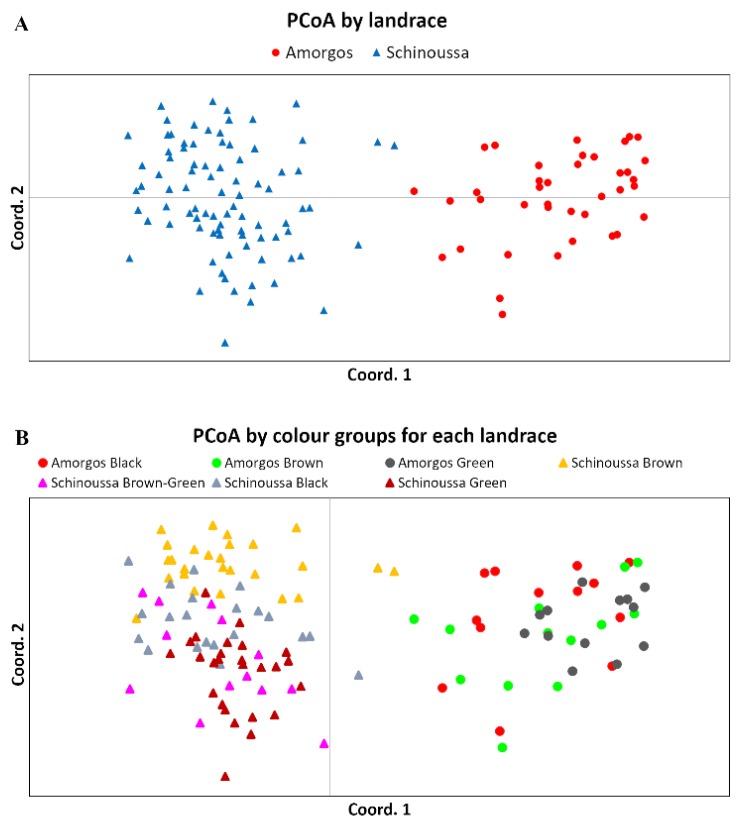
Principal Coordinates Analysis (PCoA) of the Amorgos and Schinoussa pea populations, clustering for: (**A**) population, and (**B**) seed coat color from each landrace. The PCoA analysis shows the separation of the two populations as distinct landraces based on their region of origin.

**Figure 3:**
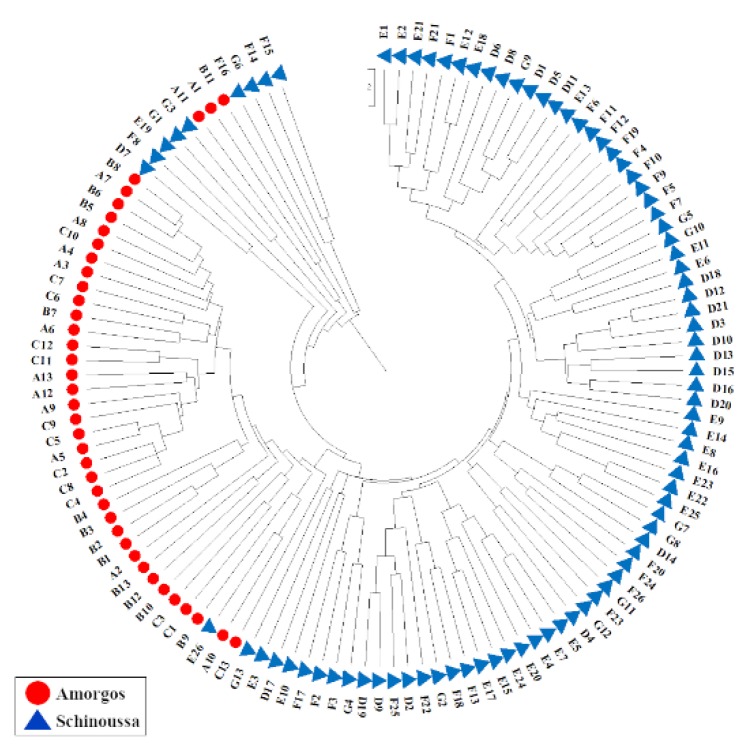
Dendrogram of the Amorgos and Schinousa pea populations based on the UPGMA analysis of the ISSR polymorphisms. Individuals are shape- and color-coded based on their region of origin (Red circle: Amorgos, Blue triangle: Schinoussa).

The Principal Coordinates Analysis (PCoA) generated two major clusters, in which samples from Amorgos and Schinoussa were clearly separated (Figure 2A). Additionally, the clustering based on seed coat color supports that the two landraces present genetic differences (Figure 2B), given Amorgos’ Black, Brown and Green peas do not overlap with the corresponding colors of Schinoussa peas, supporting the hypothesis that the Amorgos and Schinoussa pea populations are two distinct landraces. The UPGMA dendrogram illustrates that the pea samples originating from the same geographic location are closely clustered (Figure 3).

### 2.3. Molecular Screening for Powdery Mildew Using HRM Analysis

**Figure 4:**
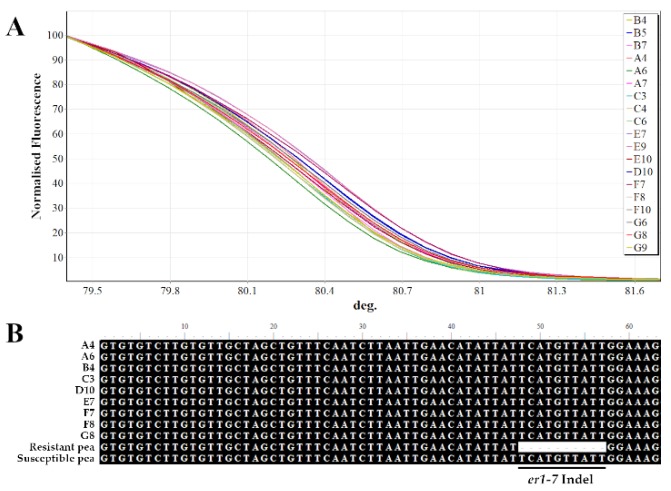
HRM analysis coupled with a co-dominant functional marker specific for *er1-7.* (**A**) Normalized fluorescence graph of selected pea samples per seed coat color and landrace. In the x axis deg. indicates temperature in °C. (**B**) Sequence alignment of the *er1-7* region from selected pea samples and the corresponding reference sequences obtained from Sun et al. (2016) [50].

HRM analysis using a specific functional marker was employed to characterize the two landraces for the presence of the powdery mildew resistance gene. Regarding the specific InDel marker, all the pea samples tested were found negative to the resistant allele (*er1-7*), across both landraces (Figure 4A). This was also confirmed by sequencing of selected samples, where the resistant allele was absent and therefore the 10-bp sequence TCATGTTATT was present (Figure 4B).

## 3. Discussion

Exploiting genetic diversity from local traditional landraces is fundamental for conservation practices and breeding programs, especially under the pressure posed for adaptation to climate change worldwide. To promote the local pea landraces of the Cyclades we aimed at identifying the species and understanding the genetic relationship of the local Amorgos and Schinousssa pea populations, using DNA barcoding and ISSR marker analysis. Over the centuries, from the prehistoric times and the Bronze Age (3000–1100 B.C in Greece) to the mid-19th century, the Aegean was a field of pirate action [51,52]. Oral traditions and place names, throughout the history of Schinoussa, suggest that the island was used as a ground and shelter for pirates. One of our hypotheses was that the landrace of Schinoussa belongs to *P. abyssinicum*, however, our results strongly suggest otherwise. Although both landraces showed a geographical clustering according to the PCoA and the UPGMA analysis of the ISSR polymorphisms, both were identified based on DNA Barcoding as *P. sativum* subsp. *elatius*.

Herein, the inability of most of the barcoding markers to discriminate samples at the species level, being not variable enough to resolve phylogeny of the genus, is probably due to the conserved chloroplast sequences and the low mutation rate [53]. The inability to discriminate among the *P. abyssinicum*, *P. sativum* subsp. *elatius* and *P. fulvum* is in agreement with the view that *P. abyssinicum* is an ancient hybrid of the two species [14]. Nevertheless, despite the inability of the *psbA-trnH* spacer on identifying species due to the frequently observed intraspecific inversions [54], in this study it was shown as the most effective marker for separating the two pea landraces from *P. abyssinicum*. Additionally, although the *matK* barcode showed the lowest resolution capacity in our study, it was sensitive enough to discriminate the two pea landraces from *P. fulvum*. Thus, the most informative barcoding markers to draw conclusions concerning the species identification in the two populations is the combination of *psbA-trnH* and *matK*. Taking into consideration the phylogenetic trees and barcoding results from all the studied markers, the two landraces are more closely related to *P. sativum* subsp. *elatius*, than to the *P. abyssinicum* and *P. fulvum* species.

There is a limited number of studies on *Pisum* germplasm that mainly are focusing on SSR analysis [55]. Herein, the ISSR analysis showed that the populations were distinguished into geographical regions, as separated clusters, indicating the adaptation of these traditional landraces to relatively different agro/climatic conditions (Figure 2 and Figure 3). This geographical isolation could have potentially resulted to the genetic drift of the two landraces. This result is in accordance with the larger molecular differences detected between pea landraces collected in Maritime areas of Spain [26]. Pea is known as self-pollinating with occasional cross-pollination which allows spontaneous hybridization [56]. As a self-pollinated crop, higher genetic diversity is expected among cultivars than within cultivars. However, the AMOVA analysis showed larger genetic diversity within cultivars (80%), which is in accordance with similar findings in other legumes such as chickpea [57]. This may be attributed to the natural interspecific crosses that can occur between *Pisum* species, serving as a source of additional genetic diversity for the selection of common pea [15]. This indicates that despite autogamy, the analysis of genetic diversity on some plants per landrace might be useful in breeding programs [28]. The large genetic diversity might be due to the long-term adaptation of the landraces to the local environment and the diverse agro-ecological systems [55], in combination with putative migration events among the regions, followed by introgression with pre-existing germplasms [28].

Aiming to identify unique traits in the two pea traditional landraces we screened for the powdery mildew resistance gene (*er1*). The er1 is the loss-of-function mutation in the disease susceptibility-related *PsMLO1* gene [58] and the most widespread across resistant pea germplasm, conferring penetration resistance [50,59]. HRM coupled with a functional marker has been previously reported to be highly efficient and cost-effective for routine large-scale screening of pea germplasm for resistance to powdery mildew [60]. This approach allowed for the accurate genotyping of both the homozygous and heterozygous resistant peas [58,60]. In this study, using the *er1-7* functional marker coupled with HRM, we identified that the two landraces of the Cycladic islands are susceptible to powdery mildew. These results indicate the need for further crop improvement of the traditional pea landraces by introducing resistance through molecular-assisted breeding strategies.

## 4. Materials and Methods

### 4.1. Plant Material and DNA Isolation

**Figure 5:**
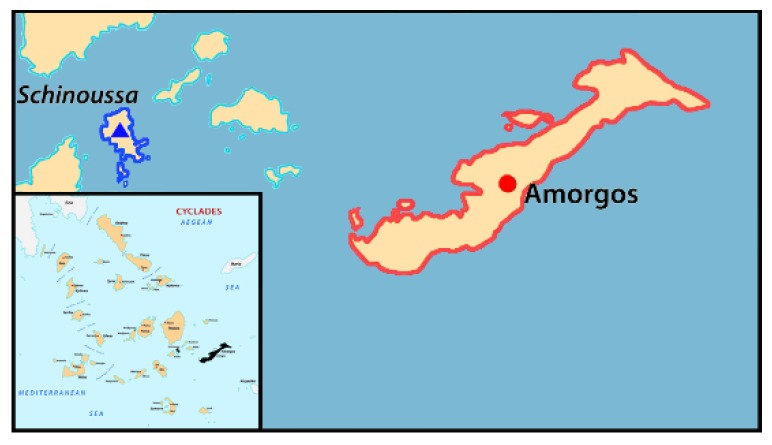
Map of the sampling sites in Amorgos and Schinoussa islands.

The plant material used in this work includes samples from two pea populations from the islands of Amorgos and Schinoussa (Table 4 and Figure 5). The pea samples were grouped based on the geographical region of origin and the seed coat color; groups A-C from Amorgos and groups D-G from Schinoussa (Table 4). Color grouping was based on the three main seed coat colors (black, brown and green) observed in both populations, except for Schinoussa, which had an additional brown-green hue (Table 4). Following, a subsample of seeds from each group was planted in pots containing 2:1 peat:perlite in order to obtain fresh leaf material for DNA extraction. Total genomic DNA was isolated from approximately 0.1 g of fresh leaf material for each sample following the modified CTAB protocol as described by Doyle and Doyle (1987) [61]. After extraction, the DNA samples were re-diluted in 1X TE buffer (10 mM Tris-Cl pH 8.0, 1 mM EDTA) and stored at −20 °C. DNA quantity and quality were assessed by regular spectrophotometric procedures using the UV-Vis Spectrophotometer Q5000 (Quawell Technology Inc., U.S.A.) and gel electrophoresis in 1% agarose gel.

### 4.2. DNA Barcoding and Sequencing Analysis

For the identification of the two landraces, we performed DNA barcoding analysis using the *ITS2* [62], *trnL* [63], *rpoC*, *matK* [35], and *psbA-trnH* [64] barcoding markers. PCR amplification was performed on a Rotor-Gene 6000 real-time 5-Plex HRM PCR Thermocycler (Corbett Research, Sydney, Australia), using the Rotor-Gene Q software version 2.0.2 (Corbett Life Science, Cambridge, UK). PCR reaction mixtures with a total volume of 20 μL consisted of approximately 20 ng genomic DNA, 1× PCR buffer, 0.5 μM forward and reverse primers, 0.2 mM dNTPs, 1.5 mM SYTO™ 9 Green Fluorescent Nucleic Acid Stain (Invitrogen, Eugene, Oregon, USA), and 1 U Kapa Taq DNA polymerase (Kapa Biosystems, USA). The universal regions were amplified using the following protocol: initial denaturation at 95 °C for 4 min, followed by 35 cycles of 95 °C for 30 sec, corresponding annealing temperature (Ta) °C for 60 sec, and 72 °C for 60 sec with a final extension phase at 72 °C for 3 min.

After sequencing, the sequences of the five candidate regions were aligned with the MUSCLE algorithm and genetic distances were calculated using Molecular Evolutionary Genetics Analysis X (MEGA X; Version 10.05) based on the K2P-distance model [65] to evaluate divergence between the two populations. The Neighbour-joining clustering method was used to demonstrate the represented differences as an unrooted dendrogram using MEGA X [65]. Statistical support for each constructed tree was provided by two statistical data analysis as bootstrapping (1000 replications) and pairwise distance model.

Species identification based on the sequence similarity approach was performed with the National Center for Biotechnology Information (NCBI) database [66] by basic local alignment search tool (BLAST; setting: blastn, megablast) [67] and all regions of the three various *Pisum* species were used as query sequences. Correct identification was concluded when the best BLAST hit of the query sequence had over 96% query coverage and identity.

### 4.3. ISSR Genotyping and Data Analysis

For the distance-based analysis of the two populations we used 15 ISSR markers of which six (UBC811, UBC818, UBC827, UBC841, UBC873, UBC880) were selected for further analysis based on their discrimination efficiency. The total volume of PCR reaction was 25 μL containing 1X PCR buffer, 0.2 mM dNTPs, 10 μM primer, 1 U/μL Taq DNA Polymerase (Kapa Biosystems Ltd.) and 20 ng template DNA. The profile of the PCR reaction program was an initial denaturation for 4 min at 94 °C, followed by 35 cycles at 94 °C for 30 s, 40 sec annealing at the corresponding Ta °C, and 40 sec extension at 72 °C, ending with final extension phase at 72 °C for 7 min. The amplified PCR products were run on 1.5% agarose gel with 1X TAE buffer at 100 V and visualized with the UV Minibis Pro (DNR Bio-Imaging Systems, Jerusalem, Israel) instrument. Band scoring was performed using the Logger Pro 3.15 software.

DNA fragment profiles were scored in a binary fission with ‘0′ indicating the absence and ‘1′ indicating presence of a band. Using the binary haploid data, a pairwise individual-by-individual genetic distance matrix was constructed using the Jaccard coefficient. The percentage of polymorphic loci (P), Number of alleles (N), Number of different alleles (Na), Number of effective alleles (Ne), gene diversity (expected heterozygosity, He), Shannon’s diversity index (I), Diversity (h) and unbiased genetic distances (uh) according to [68] were subsequently calculated. The hierarchical distribution of genetic diversity among and within populations was also characterized by Analysis of Molecular Variance (AMOVA) and Principal Co-ordinate Analysis (PCoA). All of the above analyses were performed using the GenAlex 6.5 software package [69]. The Unweighted Pair Group Method based on Arithmetic Averages (UPGMA) clustering analysis for analyzing the similarity estimates was performed using MEGA X [65] and expressed as a dendrogram.

### 4.4. Molecular Screening for Powdery Mildew Resistance Using HRM Analysis

We used a co-dominant functional marker specific for *er1-7*, the InDel111–120, associated with pea resistance to powdery mildew [50,59,60]. Representative samples were selected from each color group of both populations. The total volume of PCR reaction was 20 μL containing 1X PCR buffer, 0.2 mM dNTPs, 10 μM of the er1-7 primer, 1 U/μL Taq DNA Polymerase (Kapa Biosystems Ltd.) and 20 ng template DNA. PCR conditions where: preheating for 5 min and initial denaturation at 95 °C, followed by 35 cycles at 94 °C for 30 s transition, 30 sec annealing at 56 °C. The fluorescence was measured at the end of each extension step during the PCR cycles. The HRM was performed by an initial pre-melt conditioning of the PCR products at 95 °C for 5 sec and 50 °C for 30 sec, followed by a melt at range of 75–85 °C in increments of 0.1 °C every 2 sec. Fluorescence was measured at the end of each increment.

## 5. Conclusions

This work represents the first investigation focused on the molecular characterization of pea traditional accessions collected from the semi-arid region of Lesser Cyclades with the main goal to evaluate their genetic diversity and their population structure. Based on the DNA Barcoding and the ISSR marker analysis: i) both landraces have been identified as *P. sativum* subsp. *elatius* germplasm and ii) the landraces show a tendency for differentiation, which is in accordance with the geographical distribution of the genetic structure as an underlying evolutionary process. However, further research and phylogenetic analysis is required with larger population sizes for better understanding of the evolutionary processes that led to these differences, as well as for the preservation of existing diversity in *ex-situ* collections.

## Figures and Tables

**Table 1:** Band patterns of the Amorgos and Schinoussa populations resulted from the ISSR analysis.

Population	Number of Bands	Number of Band Frequency (> = 5%)	Number of Unique Bands
Amorgos	61	59	0
Schinoussa	66	66	5

**Table 2:** Mean value and standard error over loci for Amorgos and Schinoussa populations.

Population		N	Na	Ne	I	h	uh
Amorgos	Mean	39	1.742	1.403	0.373	0.244	0.250
	SE	0.000	0.073	0.043	0.031	0.022	0.023
Schinoussa	Mean	86	2.000	1.483	0.443	0.289	0.292
	SE	0.000	0.000	0.042	0.026	0.020	0.021

Mean = Mean value, SE = Standard error, N = Number of alleles, Na = Number of different alleles, Ne = Number of effective alleles = 1/(p^2 + q^2), I = Shannon’s Information Index = −1* (p * Ln (p) + q * Ln(q)), h = Diversity = 1 − (p^2 + q^2), uh = Unbiased diversity = (N/(N − 1)) * h.

**Table 3:** AMOVA analysis of the Amorgos and Schinoussa pea populations.

Source	Df	SS	MS	Est. Var.	%
Among populations	1	133.042	133.042	2.307	20
Within populations	123	1133.326	9.214	9.214	80
Total	124	1266.368		11.521	100

Df = Degrees of freedom, SS = Sum of Squares, MS = Mean Square, Est. Var. = Estimated Variance.

**Table 4:** Samples from Amorgos and Schinoussa pea populations used in this work. The samples were grouped in sub-groups (A–G) according to region of origin and seed coat color.

Sample Group	Number of Individuals	Region	Seed Coat Color
A	13	Amorgos	Black
B	13	Amorgos	Brown
C	13	Amorgos	Green
D	21	Schinoussa	Black
E	26	Schinoussa	Brown
F	26	Schinoussa	Green
G	13	Schinoussa	Brown-Green

## Supplementary Materials

The following are available online at https://www.mdpi.com/2223-7747/9/4/479/s1, Table S1: Pairwise distance and diversity analysis within and between populations of the five barcodes, Figure S1: Sequence alignments of the *psbA-trnH* region, Figure S2: Sequence alignments of the *ITS2*, *trnL*, *rpoC* and *matK* regions from selected pea samples and the corresponding Pisum reference sequences available in the NCBI, Figure S3: The *ITS2*, *trnL*, *rpoC* and *matK* dendrograms illustrating the phylogenetic relationships between Amorgos (red circle) and Schinoussa (blue triangle) pea populations.
