# Supplementary material for: DNA Fingerprinting and Species Identification Uncovers the Genetic Diversity of Katsouni Pea in the Greek Islands Amorgos and Schinoussa
Source: Plants (Basel). 2020 Apr 9;9(4):479. doi: 10.3390/plants9040479 (PMC7238155; doi:10.3390/plants9040479)
Supplement: Supplementary file 1 [file plants-09-00479-s001.pdf]

Article

DNA fingerprinting and species identification uncovers the genetic diversity of Katsouni pea in the Greek islands Amorgos and Schinoussa

Evangelia Stavridou [1], Georgios Lagiotis [1], Lefkothea Karapetsi [1], Maslin Osathanunkul [2,3] and Panagiotis Madesis[1,*]

[1] Institute of Applied Biosciences, Centre for Research and Technology, Thermi, GR-570 01 Thessaloniki, Greece; estavrid@certh.gr (E.S.); glagiotis@certh.gr (G.L.); lefki8@certh.gr (L.K.)

[2] Department of Biology, Faculty of Science, Chiang Mai University, Chiang Mai 50200, Thailand; maslin.cmu@gmail.com

[3] Research Center in Bioresources for Agriculture, Industry and Medicine, Chiang Mai University; Chiang Mai 50200, Thailand

* Correspondence: pmadesis@certh.gr

Table S1. Pairwise distance and diversity analysis within and between populations of the five barcodes.

Barcoding regions	*ITS2*	*trnL*	*matK*	*rpoC*	*psbA-trnH*
**Amorgos distance**	0.0013 ± 0.0012	0.0001 ± 0.0009	0.0137 ± 0.0049	0.0009 ± 0.0008	0.0021 ± 0.002
**Schinoussa distance**	0.0011 ± 0.0008	0.0007 ± 0.0007	0.0035 ± 0.0015	0.0007 ± 0.0006	0.0021 ± 0.002
**Interpopulation distance**	0.0016 ± 0.0014	0.0008 ± 0.0008	0.0086 ± 0.0028	0.0007 ± 0.0006	0.0018 ± 0.0018
**Diversity within populations**	0.0012 ± 0.001	0.0009 ± 0.0008	0.0086 ± 0.0028	0.0008 ± 0.0007	0.0021 ± 0.0021
**Diversity between populations**	0.0002 ± 0.0002	0.0000	0.0000	0.0000	0.0000
**Overall Diversity**	0.0014 ± 0.001	0.0008 ± 0.0008	0.0062 ± 0.0018	0.0007 ± 0.0007	0.002 ± 0.002

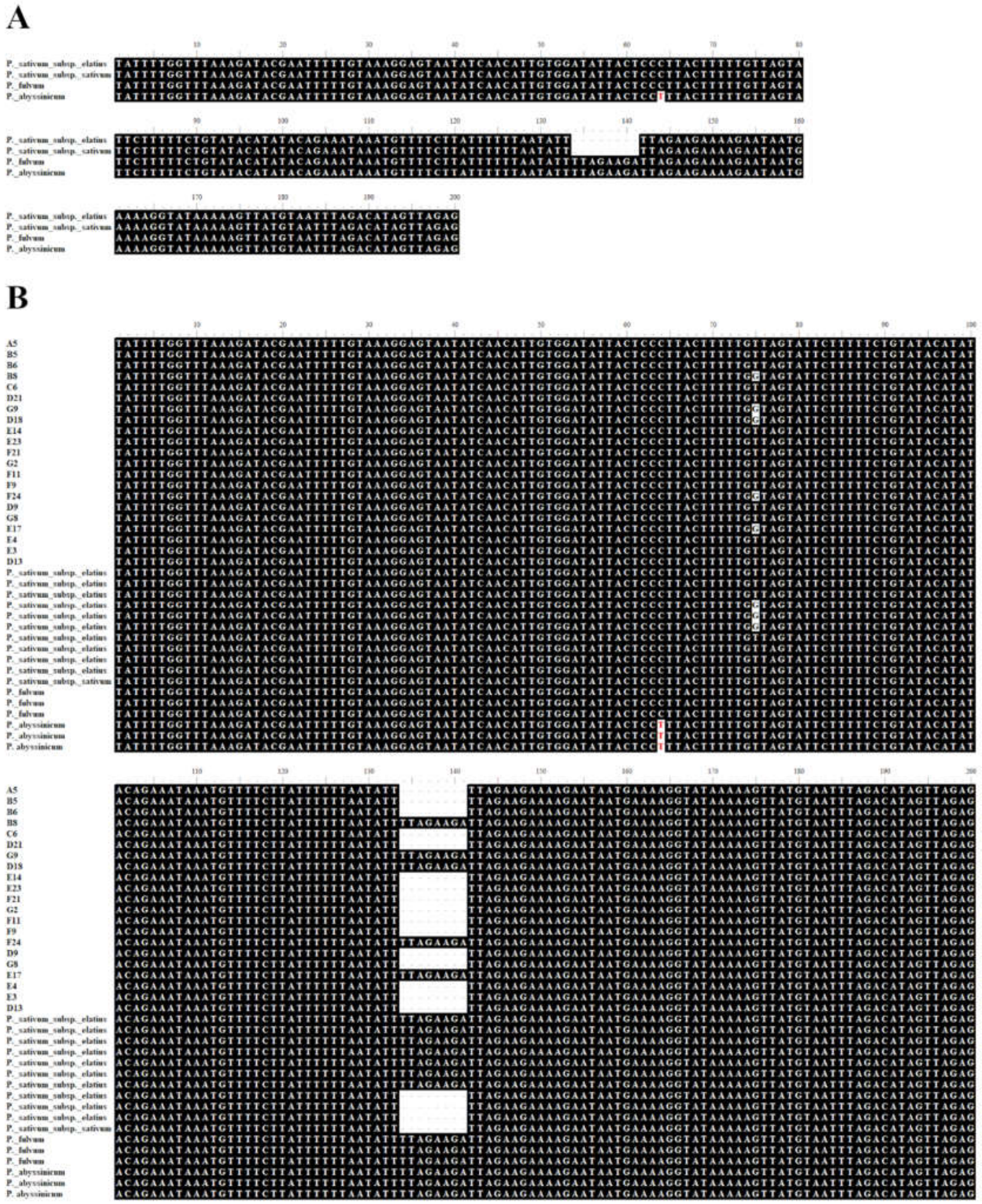

Figure S1. Sequence alignments of the *psbA-trnH* region. A) reference sequences of the *P. sativum* subsp *elatius*, *P. sativum* subsp *sativum*, *P. fulvum* and *P. abyssinicum*; B) selected pea samples and the corresponding *Pisum* reference sequences available in the NCBI. The reference NCBI accessions are in descending order the following: *P. sativum* subsp *elatius* [NC039371.1, MG882489.1, MG882488.1, LT59666.2, LT59666.1, LT59659.1, LT59667.1, HE601759.1, LM651156.1, LM651155.1]; *P. sativum* subsp *sativum* [HG966674.1]; *P. fulvum* [FR856870.1, FR856866.1, FR856873.1]; and *P. abyssinicum* [LT596675.1, FR856867.1, LN876624.1].

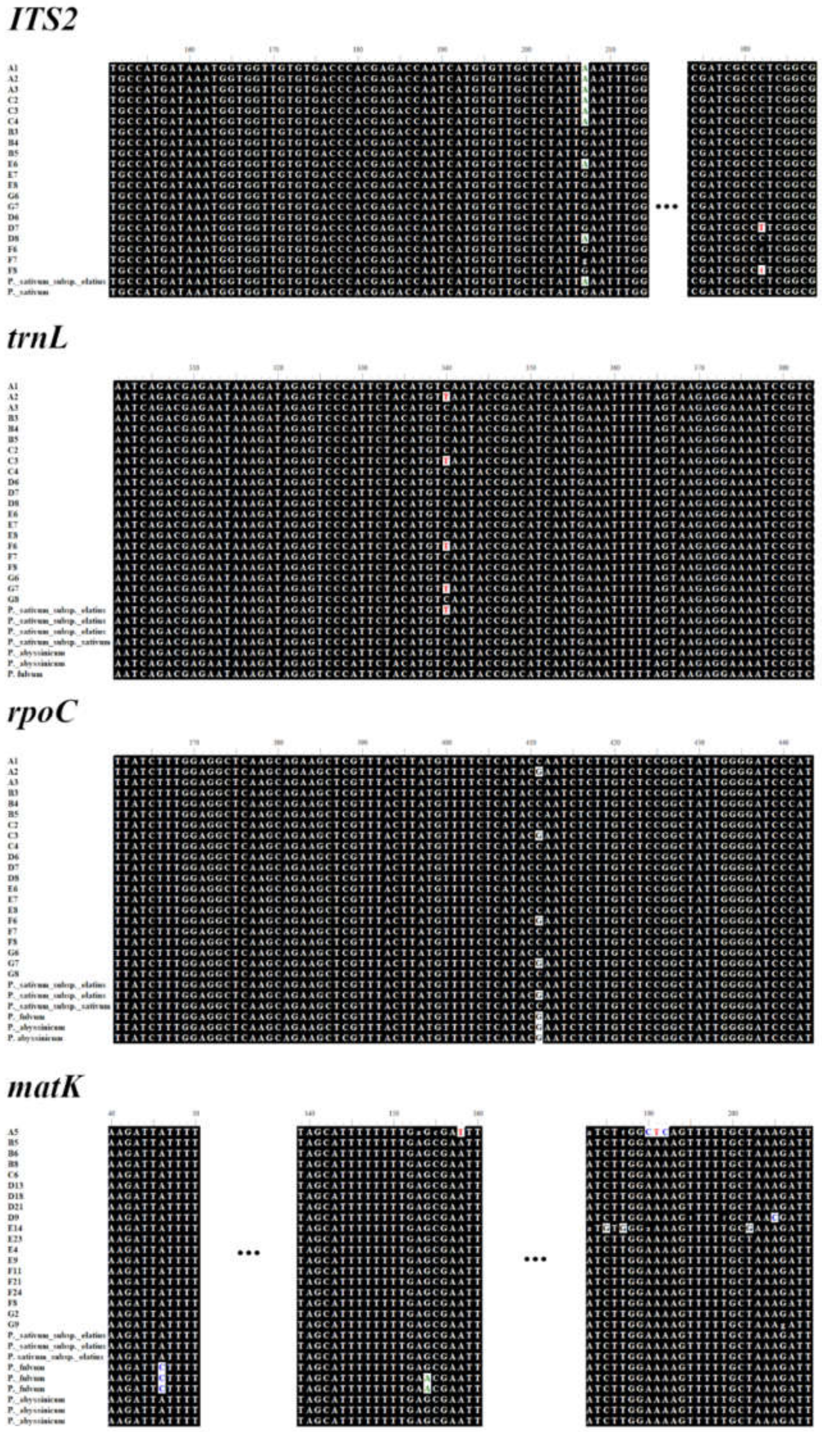

Figure S2. Sequence alignments of the *ITS2*, *trnL*, *rpoC* and *matK* regions from selected pea samples and the corresponding *Pisum* reference sequences available in the NCBI. The reference NCBI accessions are in descending order the following: ***ITS2*** [JN617189.1, JN617190]; ***trnL*** [MG859921.1, MG882488.1, NC039371.1, MG917089.1, MG859923, NC037830.1, LC311178.1]; ***rpoC*** [JN617139, NC039371.1, MG917089.1, MG458702.1, MG859923.1, NC037830.1]; and ***matK*** [JX677854.1, MG882489.1, NC039371.1, JX677853.1, MG458702.1, MG458703.1, MG859923.1, JX677851.1, NC037830.1].

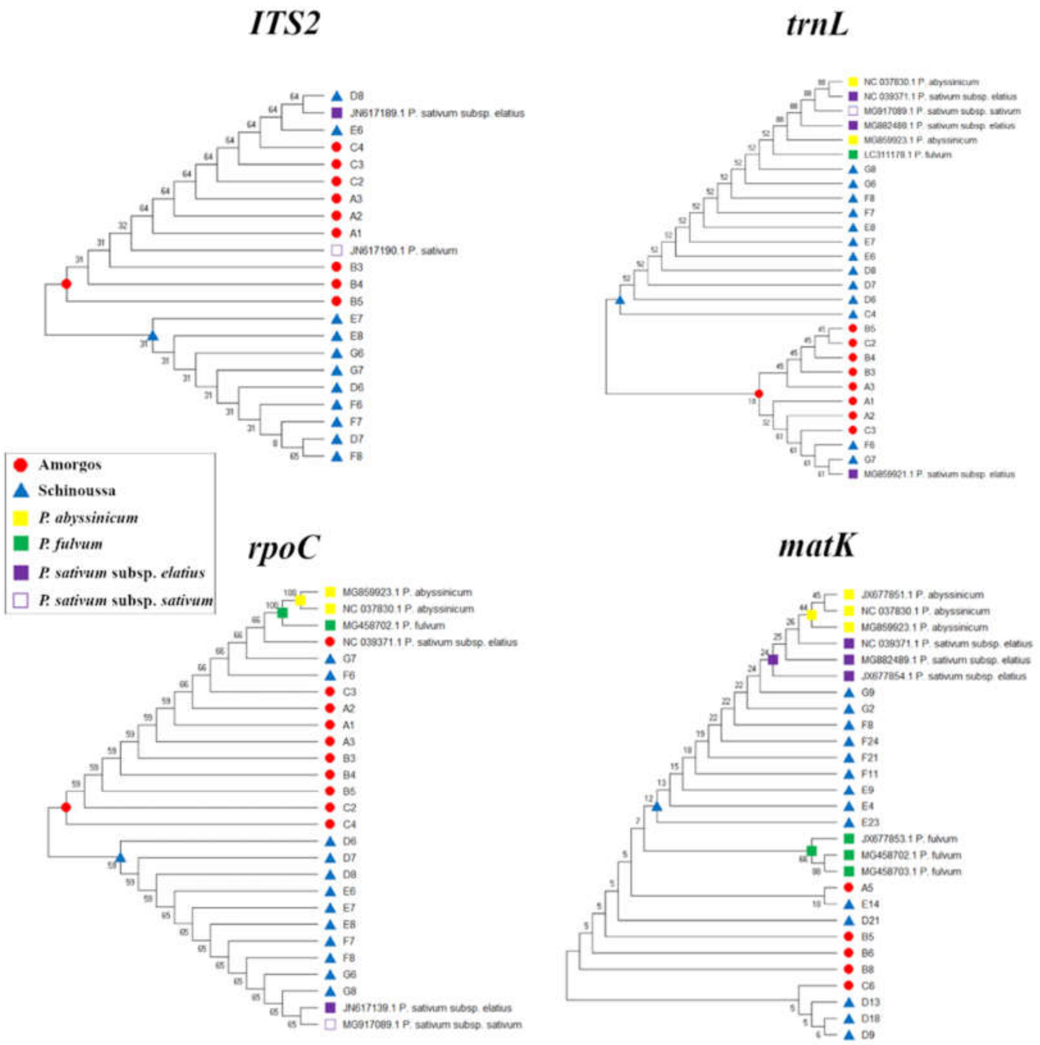

Figure S3. The *ITS2*, *trnL*, *rpoC* and *matK* dendrograms illustrating the phylogenetic relationships between Amorgos (red circle) and Schinoussa (blue triangle) pea populations. The corresponding NCBI sequences of the *P. sativum* subsp. *elatius* (purple square), *P. sativum* subsp. *sativum* (white square), *P. abyssinicum* (yellow square) and *P. fulvum* (green square) were used as reference taxa.
